# Comparison of acridine orange fluorescent microscopy and gram stain light microscopy for the rapid detection of bacteria in cerebrospinal fluid

**DOI:** 10.1186/s13104-020-4895-7

**Published:** 2020-01-13

**Authors:** Supriya Sharma, Jyoti Acharya, Megha Raj Banjara, Prakash Ghimire, Anjana Singh

**Affiliations:** 1 Central Department of Microbiology, Tribhuvan University, Kirtipur, Kathmandu, Nepal; 2 National Public Health Laboratory, Teku, Kathmandu, Nepal

**Keywords:** Bacterial meningitis, Culture, Acridine orange stain, Gram stain

## Abstract

**Objective:**

Bacterial meningitis is a life threatening condition that requires prompt recognition and treatment. Currently, Gram stain is widely used for the microscopic detection of bacterial pathogens in cerebrospinal fluid (CSF). In Nepal, fluorescent microscopes have been installed in laboratories as a part of the National tuberculosis control program. However, information on the utility of the acridine orange (AO) stain for the direct detection of bacteria in CSF samples in Nepal is not available. Therefore, this study aims to compare Gram stain and AO stain for the rapid detection of bacterial pathogens in CSF of clinically suspected meningitis cases in Kathmandu, Nepal.

**Results:**

Bacterial pathogens were detected in 9.30% (36/387) by either of the three tests, 9.04% (35/387) by AO stain, 8.27% (32/387) by culture and 6.46% (25/387) by Gram’s stain. Considering culture as a gold standard, the sensitivity of AO stain was higher than Gram stain. The specificity of AO stain was 98.87%. Detection and differentiation of the bacteria was much clear in AO staining than Gram staining. AO is a better alternative to Gram stain in the rapid detection of bacterial pathogens in CSF in the setting where fluorescent microscope is available.

## Introduction

Bacterial meningitis is a life threatening condition that requires prompt recognition and treatment [1]. The case fatality rate without treatment may be 70%. About 20% of survivors of bacterial meningitis may be left with permanent sequelae such as hearing loss, neurologic disability, or loss of a limb [2]. World Health Organization reported around one million suspected meningitis cases (1991–2010) among countries of the African Meningitis Belt [3]. A rapid diagnosis is fundamental for decreasing morbidity and mortality from bacterial meningitis. The laboratory diagnosis of bacterial meningitis includes direct visualization of bacteria through microscopy, culture and/or the detection of bacterial antigens in the cerebrospinal fluid (CSF). Though culture is relatively slow, it remains the gold standard. Antigen detection is rapid but is not always available in low resource settings [4]. Therefore, microscopy remains a rapid, cheap and reliable diagnostic method.

Currently Gram stain is widely used for the microscopic detection of bacterial pathogens in CSF [2]. Gram stain is usually reliable at detecting > 10^5^ bacteria per millilitre of the body fluid [5]. The CSF specimens stained with Gram stain must be observed carefully since only few poorly stained bacteria might be present on the whole slide and inflammatory cells, erythrocytes and precipitated stain may mask the bacteria. Therefore, stains other than Gram stain like acridine orange (AO) stain can be used to screen CSF smears for bacteria.

AO, a fluorochrome stain, has the ability to intercalate into nucleic acid. The bacteria appear to be bright red while leukocytes appear pale apple green at low pH (4.0). Literature suggests that AO stain is more sensitive than Gram stain. It has the ability to detect bacteria at > 10^4^ CFU/ml concentrations which is about tenfold lower than that detectable by the Gram stain [6]. Moreover, the time devoted for examining CSF smear is reduced due to the remarkable contrast between the brightly stained bacteria and the dark background [5]. AO stain requires fluorescent microscope. In Nepal, fluorescent microscopes have been installed in the laboratories of major hospitals as a part of National Tuberculosis Control Program of Nepal (personal communications). Information on the utility of the AO stain for the direct detection of bacteria in CSF samples in Nepal is not available. Therefore, this study aims to compare the Gram stain and AO stain for the rapid detection of bacterial pathogens in the CSF of clinically suspected meningitis cases in Kathmandu, Nepal.

## Main text

### Methods

#### Study design

This prospective cohort study was conducted from January 2017 to December 2018 among 387 CSF samples collected from clinically suspected meningitis cases attending different hospitals located at Kathmandu, Nepal. The details on study population and specimen collection have been described elsewhere [7]. Each specimen was processed for culture by conventional bacteriological techniques at the respective collection sites. Gram’s staining and AO staining of each specimen was done at National Public Health Laboratory, Teku, Kathmandu, Nepal.

#### Gram’s staining

Air-dried, heat-fixed smear prepared from each CSF sample (50 µl) was stained sequentially with crystal violet solution (Hi Media) for 1 min, iodine solution (Hi Media) for 1 min and decolorized by acetone–alcohol (Hi Media) for 20 s and counterstained with safranin solution (Hi Media) for 1 min. The slide was then observed under light microscope (Olympus) under oil immersion at 1000×.

#### AO staining

The AO stain (100 mg/litre) (Sigma-Aldrich) was prepared as previously described [6]. The solution was filtered through 0.22 µm pore size filter. The staining solution was then stored in a brown bottle at room temperature [8]. Smear was prepared from each CSF sample (50 µl), air dried and fixed with methanol for 1 min. It was then flooded with the AO stain for 2 min, washed with water, dried, and examined under 100× and confirmed under oil immersion at 1000× with an epi-illumination fluorescence microscope (FITC filter). Each batch of the AO and Gram stain was quality controlled with smears of *Escherichia coli* ATCC 25922 and *Staphylococcus aureus* ATCC 25923.

#### Culture

Isolation and identification of bacterial pathogens from CSF samples was done by conventional culture techniques. The details are described elsewhere [7].

#### Data analysis

The obtained data was entered into Microsoft office Excel 2007. Sensitivity, specificity, positive predictive value (PPV) and negative predictive value (NPV) of Gram stain and AO stain was calculated considering culture as a gold standard test. Sensitivity, specificity, PPV and NPV was calculated as:

$$ {\text{Sensitivity}}\, = \,{\text{TP}}/\left( {{\text{TP}}\, + \,{\text{FN}}} \right) \, \times { 1}00 $$

$$ {\text{Specificity}}\, = \,{\text{TN}}/\left( {{\text{TN}}\, + \,{\text{FP}}} \right) \, \times { 1}00 $$

$$ {\text{Positive predictive value}}\, = \,{\text{TP}}/\left( {{\text{TP}}\, + \,{\text{FP}}} \right) \, \times { 1}00 $$

$$ {\text{Negative predictive value}}\, = \,{\text{TN}}/\left( {{\text{TN}}\, + \,{\text{FN}}} \right) \, \times { 1}00 $$

where TP is true positive, FP is false positive, TN is true negative and FN is false negative. A *P* value of < 0.05 was considered to be statistically significant.

### Results

Bacterial pathogens were detected in 9.30% (36/387) by either of the three tests, 9.04% (35/387) by AO staining, 8.27% (32/387) by culture and 6.46% (25/387) by Gram’s staining. Only 6.46% of the samples were positive for all the three tests i.e. Gram’s stain, acridine orange stain and culture. The sensitivity of AO stain was higher than Gram stain. The specificity of AO stain was 98.87% (Table 1).

**Table 1:** Comparison of Gram and AO stain in the direct microscopic examination of CSF specimens (n = 387)

Gram stained smear microscopy results versus culture results						
Gram’s stain	Culture		Sensitivity	Specificity	Predictive value	
	Pos	Neg			Pos	Neg
Pos	25	0	78.12%	100%	100%	98.06%
Neg	7	355				

AO stained smear microscopy results versus culture results						
AO stain	Culture		Sensitivity	Specificity	Predictive value	
	Pos	Neg			Pos	Neg
Pos	31	4	96.88%	98.87%	88.57%	99.70%
Neg	1	351				

Correlation between the Gram and AO staining of the same sample was good (Spearman = 0.833). The difference in positivity (7 specimen missed by Gram stain) between the two staining procedures was statistically significant (p value < 0.001). Of the 7 AO positive and Gram stain negative specimens, 6 were culture positive for bacterial pathogens (Table 2).

**Table 2:** Type of bacteria missed by Gram stain but detected by AO stain (n = 6)

Bacteria	Number	Percentage
*Escherichia coli*	1	16.67
*Haemophilus influenzae*	3	50.00
*Neisseria meningitidis*	2	33.33

**Fig. 1:**
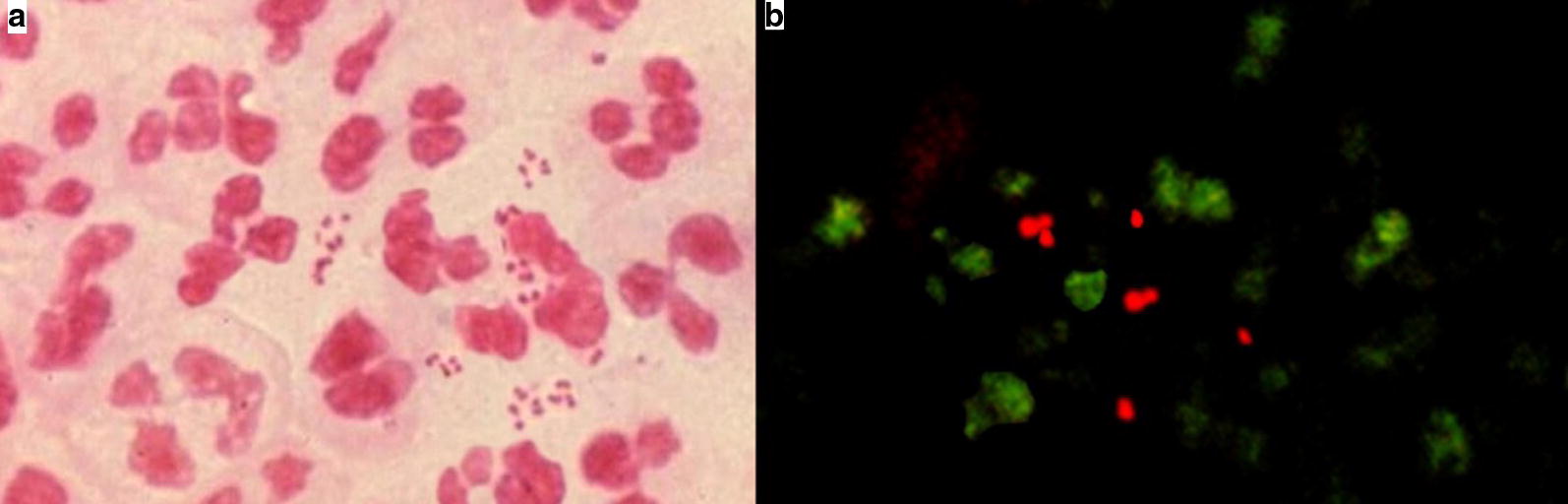
CSF smear stained with Gram’s stain and acridine orange stain. **a** Image of a positive Gram’s stain slide showing pink coloured Gram negative diplococci bacteria. **b** Image of a positive acridine orange stained slide showing bacteria fluorescing orange red against a dark background using a blue filter on a fluorescent microscope

Detection and differentiation of the bacteria was much clear in AO stained smear than Gram stained smear (Fig. 1). Excluding the cost of fluorescent microscope, both AO stain and Gram’s stain have similar price.

### Discussion

The rapid detection of pathogens in CSF is central to the diagnosis and treatment of meningitis cases. Gram’s stain and culture are routine methods for laboratory diagnosis of bacterial meningitis in Nepal. However, AO stain has been widely used for the detection of microorganisms in direct smears of clinical specimens including CSF in developing and developed countries [9–14].

AO stain was more sensitive than the Gram stain in our study. Studies have reported the sensitivity of Gram stain between 40 and 93% [15–18]. The sensitivity of AO in this study was similar to that reported by Neeraja et al. [10]. In contrast, much lower sensitivity of AO has been reported in other previous studies [6, 19]. The sensitivity of AO and Gram’s stain was reported to be equivalent in some studies [9]. Grando et al. [20] concluded that there was no clear advantage of AO stain instead of Gram’s stain in case of ocular specimens. AO staining produces bright orange fluorescing bacteria against dark background which is pleasing to the eye unlike with the Gram’s stained smear [6]. Therefore, there might remain possibility of missing the darkly stained bacteria against light background in Gram’s stained smear. However, Gram’s stain has an extra advantage of distinguishing between Gram negative and Gram positive bacteria whereas AO stain only detects the bacteria.

In the present study, AO proved to be particularly helpful in the early detection of Gram negative bacteria which were otherwise missed by Gram’s stain. Neeraja et al. [10] have reported the utility of AO in the early detection of candidaemia . In four specimens, bacterial pathogens were detected by AO stain but negative by culture in our study. This might be false negative culture results due to the fastidious nature of the bacteria or false positive AO stain results due to the other obscuring materials in the smear. Therefore, the specimens should be reconfirmed with other alternative test such as PCR to confirm the result. Inclusion of ultimate diagnoses of apparently negative cases would help mitigate concerns that even AO is insufficient to help a major diagnostic issue. Further studies should be done regarding the modification of AO staining. Previously, few researchers have utilized AO along with other stains in the differential fluorescent staining method for the detection of bacteria in CSF and other clinical specimens. The microorganisms were easily detected, even when bacterial counts in the specimens were low [21]. Researchers have also shown the use of acridine orange as an indicator of bacterial susceptibility to gentamicin [22] AO stain should be modified by combining it with other stains so that it will not only detect bacteria but also be able to differentiate gram positive and gram negative bacteria at the same time.

Seven culture positive cases were detected as negative by Gram’s stain in our study. In contrast, studies have shown additional value of Gram staining of CSF in culture negative cases. Gram staining was less affected by antibiotic presence and might be useful when antibiotics were previously administered [15, 16]. To the best of our knowledge, this is the first study in Nepal to compare AO with Gram stain for the detection of bacteria in CSF. Though culture is considered as gold standard test for the diagnosis of bacterial meningitis, specimens might have been falsely categorized as negative due to the fastidious nature of bacteria. Furthermore, the yield of culture might have been decreased as 58% of cases were referred from other health care settings and pre-treated with antibiotics. In culture negative meningitis cases, PCR allows for the definitive identification of bacteria. However, cost remains a barrier for molecular diagnostics in developing countries [23].

### Conclusion

AO is a better alternative to Gram stain in the rapid detection of bacterial pathogens in CSF in the setting where fluorescent microscope is available. We recommend, however, that positive smears be reexamined with the Gram stain to determine the Grams reaction of the bacteria.

## Limitations

The major limitation of our study is that there were only few culture positive specimens. Such specimens should further be tested with alternative methods like PCR to confirm the results.

## Data Availability

All data generated or analysed during this study are included in this published article.

## Abbreviations

CSF: cerebrospinal fluid
AO: acridine orange
PCR: polymerase chain reaction
